# Gender differences in the intention to withhold life-sustaining treatments involving severe dementia for self and on behalf of parent or spouse

**DOI:** 10.1186/s12904-022-01062-8

**Published:** 2022-10-06

**Authors:** Duan-Rung Chen, Jih-Shuin Jerng, Daniel Fu-Chang Tsai, Yuchi Young

**Affiliations:** 1 Institute of Health Behaviors and Community Sciences, National Taiwan University, Taipei, Taiwan; 2 Department of Internal Medicine, National Taiwan University Hospital, Taipei, Taiwan; 3 Department & Graduate Institute of Medical Education and Bioethics, National Taiwan University College of Medicine, Taipei, Taiwan; 4 Department of Health Policy, Management & Behavior, School of Public Health, New York State University,, Albany, USA

**Keywords:** Gender differences, Life-sustainment treatments, The patient autonomy act

## Abstract

**Background:**

Few studies have explored gender differences in the attitudes toward advanced care planning and the intention to withhold life-sustaining treatments (LSTs) involving severe dementia in Asian countries. We examined gender differences in the attitude toward the Patient Autonomy Act (PAA) in Taiwan and how the gender differences in these attitudes affect the intention to withhold LSTs for severe dementia. We also investigated self–other differences in the intention to withhold LSTs between genders.

**Methods:**

Between March and October 2019, a structured questionnaire was distributed to hospitalized patients’ family members through face-to-face contact in an academic medical center. Exploratory factor analysis and independent and paired-sample *t-tests* were used to describe gender differences. Mediation analyses controlled for age, marital status, and education level were conducted to examine whether the attitude toward the PAA mediates the gender effect on the intention to withhold LSTs for severe dementia.

**Results:**

Eighty respondents filled out the questionnaire. Exploratory factor analysis of the attitude toward the PAA revealed three key domains: regarding the PAA as (1) promoting a sense of abandonment, (2) supporting patient autonomy, and (3) contributing to the collective good. Relative to the men, the women had lower average scores for promoting a sense of abandonment (7.48 vs. 8.94, *p* = 0.030), higher scores for supporting patient autonomy (8.74 vs. 7.94, *p* = 0.006), and higher scores for contributing to the collective good (8.64 vs. 7.47, *p* = 0.001). Compared with the women, the men were less likely to withhold LSTs for severe dementia (15.84 vs. 18.88, *p* = 0.01). Mediation analysis revealed that the attitude toward the PAA fully mediated the gender differences in the intention to withhold LSTs for severe dementia. Both men and women were more likely to withhold LSTs for themselves than for their parents. Compared with the women, the men were more likely to withhold resuscitation for themselves than for their parents (*p* = 0.05). Women were more likely to agree to enteral tube feeding and a tracheotomy for their husbands than for themselves; men made consistent decisions for themselves and their wives in those LST scenarios.

**Conclusion:**

Gender influences the attitude toward advanced care planning and consequently affects the intention to withhold LSTs, indicating that there may be a difference in how men and women perceive EOL decision-making for severe dementia in Taiwan. Further studies are warranted.

## Introduction

The treatments people choose near the end of life may differ from those they receive [1]. End-of-life (EOL) decisions are commonly arranged through advance directives (ADs), legal documents that outline treatment preferences, or by designating power of attorney to ensure that patients receive care consistent with their wishes when incapacitated. Studies highlight the benefits of ADs, with patients in the United States having ADs being less likely to die in the hospital [1–3] and more likely to receive care that is consistent with their preferences [2], and have surrogates who report more effective communication with physicians near the end of life [3]. However, many patients may have limited knowledge of future treatments, and thus they may be reluctant to complete an AD [4]. The absence of an AD may lead to the application of non-beneficial therapies that can increase suffering for patients and their families.

The Patient Autonomy Act (PAA), which was passed in Taiwan in 2019 [5], is the first relevant legislation in Asia to protect a patient’s right to natural death; it allows individuals aged ≥ 20 years to make decisions through advanced care planning, and complete a written AD to decline medical treatments in specific clinical scenarios in case of incapacity [5]. With the PAA, capable adults can provide written EOL care instructions and complete a durable power of attorney for health care. The person appointed in this document will make treatment decisions for the patent in case of incapacitation. Such requests have long been subject to legal protection in Western countries, such as that provided by the Patient Self-Determination Act passed by the US Congress in 1990 and the Mental Capacity Act passed in the United Kingdom in 2005 [6, 7]. Despite the rights afforded by the Patient Self-Determination Act, the rate of advanced care planning remains low [8]. A recent systematic review examining studies published between 2011 and 2016 indicated that only approximately 36.7% of US adults have ADs [9]. In the UK, the estimated proportion of adults with ADs is about 4% in England and just 2% in Wales, while in Germany, approximately 10% of the general population [10]. In Taiwan, less than 0.2% of the adult population (35,545 out of roughly 19.39 million adults) had completed an AD by the end of 2021 [11]. Life-sustaining treatments (LSTs) are widely used for patients approaching the end of life [12, 13], highlighting the importance of understanding the barriers to AD completion [14].

Cultural norms influence attitudes regarding patient autonomy in decisions about EOL care [15]. Individuals with positive attitudes toward patient autonomy exhibit greater willingness to sign an AD [16]; moreover, willingness to sign an AD is positively associated with older age, higher educational attainment [17], white race [3], employment, knowledge of hospice and palliative care [18], comprehensions of the illness condition [18, 19], and life satisfaction [20]. Several studies also point to sex-based gender differences in the preferences for life-sustaining treatments. For example, a qualitative study reported gender differences in attitudes concerning advance care planning, with men fearing harm from health treatments and hesitant to disclose EOL wishes to physicians. By contrast, women anticipated the benefits of ADs and believed that having one could prevent unwanted mechanical life support [21]. A study in the United States, which tracked 301 advanced cancer patients, also found that male patients are more likely to receive life-prolonging medical treatments [22]. A muti-hospital survey of 2,329 patients with advanced cancer in Taiwan found that compared with men, women were less likely to regard prolonging life as a medical goal of end-stage cancer care and were more inclined not to receive intubation and ventilator therapy [23]. Another study in the United States indicated that the decisions to withhold or withdraw potentially life-prolonging treatments are more often made in women (28.0% vs. 22.8%, P = 0.003) [24]. Men with advanced cancers are more likely than women to receive aggressive, non-beneficial ICU care near death [25]. A recent systematic review of the literature reveals that women are more likely to withdraw or withhold life-prolonging treatments than men [26]. Although empirical findings point to sex-based differences in the preferences for life-sustaining treatments, few studies discuss the underlying socialized gender roles that may affect how men and women perceive EOL decision-making.

Despite their benefits, ADs might not be available for most hospitalized patients [2]; family members serve as surrogate decision-makers in such cases. Growing evidence suggests that the decisions of surrogate decision-makers often differ from those of patients [27], probably because of psychological distance [27] or social distance [28] between the two parties. Research has revealed that surrogate decisions regarding EOL care might not benefit the patient [29], whose wishes may be compromised in such situations [30]. These decisions might differ from those the surrogate might make for themselves [31]. Family members as surrogates commonly regard their role as being the patient’s voice, an advocate for the patient, an advocate for other family members, or an advocate for themselves [32].

Surrogate decisions may also be affected by socialized gender roles. A study reported that surrogate choices in the United States are most likely to be made by daughters (58.9%) [33]. In Japan, female surrogates are more likely to change their preference from cardiopulmonary resuscitation (CPR) to do-not-resuscitate (DNR) orders than male surrogates [34]. In Chinese society, sons typically make surrogate decisions, although female family members are more likely to care for the patient [35, 36].

This study assumes that gender is socially constructed roles, behaviors, and attributes that a society considers appropriate for men and women. In Taiwan, although the gender gap has narrowed considerably in terms of educational attainment [37], the gender difference in the labor market participation rate persists (51.5% for women and 67% for men, Directorate-General of Budget, Accounting and Statistics [DGBAS], 2021). Wives, daughters, and daughters-in-law represent 63% of the caretakers of disabled or ill household members [38, 39]. A recent study revealed different disability trends for Taiwanese men and women aged ≥ 50 years, with women progressing 18% more rapidly than men toward more substantial disability after its initial onset; older age resulted in a 1.2 times faster rate of change in disability for women than for men (*p* < 0.001) [40]. Women may experience higher fatigue levels than men at the end of life [41]. Women were also less likely to regard prolonging life as a medical goal of end-stage cancer care and were more inclined not to receive intubation and ventilator therapy [23]. Therefore, Taiwanese women may be expected to follow the gender role to be family-centered and probably internalize the sense of sacrifice to reduce family burden [42]. When facing the choices about life-sustainment treatments at the end of life, they may be more likely to withhold prolonging life treatments than men.

This study aims to examine gender differences in the attitude toward the Patient Autonomy Act (PAA) in Taiwan and how the gender differences in these attitudes affect the intention to withhold LSTs for severe dementia. We also hypothesize that gender differences might exist in self–other differences in the intention to withhold LSTs involving severe dementia.

## Methods

### Study design

We conducted a quantitative survey to investigate whether men and women differ in their intention to withhold LSTs in cases involving severe dementia for themselves and on behalf of their family members. The description of severe end-stage dementia—based on the Functional Assessment Staging Test [43, 44]—was as follows: “When you have severe dementia, you do not remember many things, forget many people, cannot express your needs, or understand others, and have a deteriorating mental state. You need someone to take care of your daily tasks for an extended period; for example, you need help getting dressed, eating, and taking a bath. Gradually, you become incontinent, your mobility diminishes, and you eventually need to be in a wheelchair or stay in bed for long periods.”

After the respondents read the description, they indicated their preferences for the following five medical interventions for prolonging life: (1) receiving enteral tube feeding if severe dementia hinders eating; (2) receiving dialysis for kidney failure; (3) being intubated on a ventilator if severe dementia makes breathing impossible; (4) undergoing a tracheotomy following one month of intubation and if survival appears unlikely without a ventilator; and (5) undergoing CPR if a cardiac arrest because of end-stage dementia [45].

Scenarios involving severe dementia were selected because providing a realistic prognosis for patients with dementia is challenging [46]. Realistic forecasts are essential for patients and caregivers to prepare for subsequent difficult situations. Several reasons are listed: (1) doctors and family members do not regard advanced dementia as a terminal disease; (2) excessive focus on cognitive and physical impairments can limit palliative care options; (3) patients with dementia often present distinct illness trajectories over a long period between diagnosis and death, often without an acute deterioration [47]. Before the introduction of the PAA, most doctors in Taiwan favored active treatment of patients with dementia to ensure that the patient’s medical needs were met and to avoid legal repercussions [48].

### Study sample

This study was conducted at the National Taiwan University Hospital in Taipei, Taiwan, from March to October 2019. The data were collected through a structured questionnaire through face-to-face contact with the family members of patients treated in this hospital’s intensive care unit (ICU) and general internal medicine and surgical wards. All the participants provided written informed consent before participating in this study. This study was performed by the Declaration of Helsinki and approved by the National Taiwan University Research Ethics Committee (No. 201811035RINB).

The inclusion criteria for the interview participants were as follows: (a) related by blood or law to an in-patient who had been hospitalized for 48 h or longer, (b) visited the patient more than twice during the patient’s hospitalization (or served as the primary caregiver during the hospitalization), and (c) aged ≥ 20 years and able to answer interview questions. Potential participants were excluded if they could not speak or read Chinese fluently or could not be present at the hospital for the face-to-face interview. We assumed that recruiting family members of hospitalized patients would be more likely to reflect on matters concerning EOL decisions.

To determine the necessary number of respondents, we used the G*power program [49]. G*Power [50]was designed as a general stand-alone power analysis program for statistical tests in social and behavioral research. For studies with two to five predictors, the required sample size ranges from 68 to 92, for an effect size of 0.15, an error probability of 0.05, and a power (1 − *B* error probability) of 0.8. Therefore, we adopted a sample size of 100.

### Analysis plan

First, univariate and bivariate analyses were conducted to examine the gender differences in variables of interest in this study. Second, independent and paired-sample t-tests (for self–other differences) were used to describe the difference between men and women. Third, exploratory factor analysis and reliability tests were used to examine the respondents’ attitudes toward the PAA. Lastly, we investigated whether men and women differ in their attitudes toward the PAA and whether the differences in the attitudes affect their intention to withhold LSTs for severe dementia by using mediation models.

**Fig. 1:**
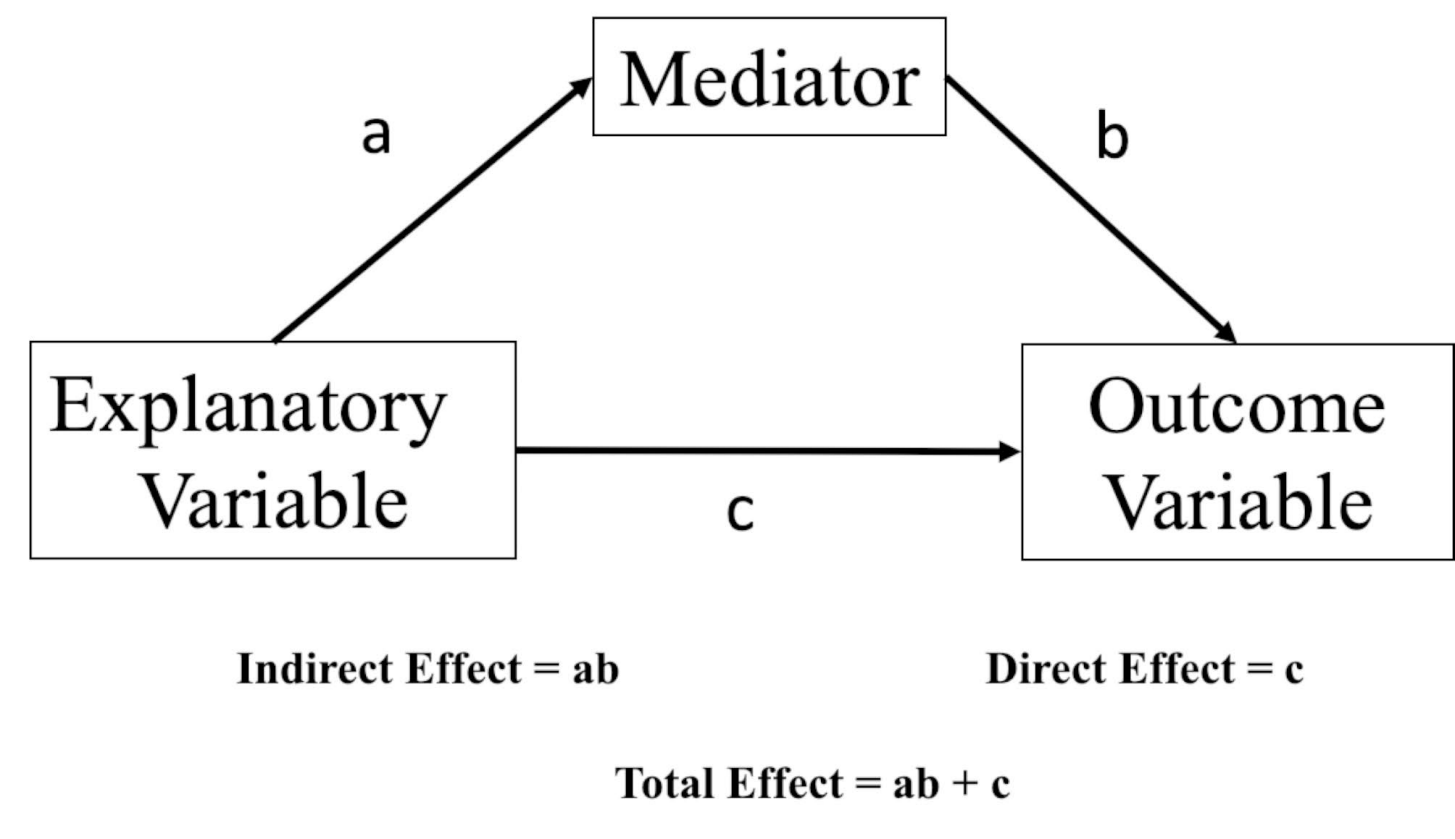
Scheme of a simple mediation model

The mediation models in Hayes’ PROCESS Macro (2022) were used to examine the direct, indirect, and total effect of gender on the intention to withhold LSTs for severe dementia (also see Fig. 1). Bootstrapping (5000 resamples) was used to estimate the 95% confidence interval (CI) for the aforementioned effects. All the statistical analyses were performed using SPSS 19.0 (SPSS, Chicago, IL, USA).

For the mediation analysis, we specifically examined (1) whether men and women differ in the intention to withhold LSTs for severe dementia (the direct effect “c”) and (2) whether men and women differ in their attitudes toward the PAA (the effect “a”), and (3) whether these attitudes toward the PAA affect the intention to withhold LSTs for severe dementia (the effect “b”). The total effect of gender is the direct effect (“c”) plus the indirect effect (“ab”) (see Fig. 1).

### Measures

Based on the previous studies [45, 51], we adopted a modified Chinese version of the Life Support Prevalence Questionnaire (LSPQ) to measure respondents’ preferences for life-sustaining treatments for severe dementia. Second, we developed a questionnaire and invited three experts to review the feasibility of its contents. The questionnaire consisted of two parts, which evaluated the following: (1) general attitude toward the PAA and (2) hypothetical LST decisions involving severe dementia for themselves and on behalf of people close to them.

### Outcome variables

Intention to Withhold LSTs for severe dementia was defined according to respondent ratings for the following five medical interventions for prolonging life: (1) receiving enteral tube feeding if severe dementia hinders eating; (2) receiving dialysis for kidney failure; (3) being intubated on a ventilator if severe dementia makes breathing impossible; (4) undergoing a tracheotomy following one month of intubation and if survival appears unlikely without a ventilator; and (5) undergoing CPR if a cardiac arrest because of end-stage dementia. Respondents rated each medical intervention on a 5-point Likert scale from 1 (*strongly agree*) to 5 (*strongly disagree*). Exploratory factor analysis was performed, and one factor with an explained variance of 54% was found. This factor has a Cronbach’s α value of 0.92. We summed the ratings of these five medical interventions, yielding scores ranging from 5 to 25: a higher score indicates a stronger intention to withhold LSTs for severe dementia.

### Explanatory variables

Gender was coded as 1 for men and 0 for women.

Attitude toward the PAA was defined according to responses to nine items on how respondents regarded the PAA. Items were rated on a 5-point Likert scale from 1 (*strongly disagree*) to 5 (*strongly agree*). Exploratory factor analysis was conducted, and three factors were identified: Factor 1, promoting a sense of abandonment, comprised four items: With the PAA, (1) I am basically waiting to die; (2) I feel insecure about my medical treatments in the future; (3) my quality of care will not be guaranteed; (4) healthcare providers will no longer care for any of my medical conditions. This factor had a Cronbach’s α value of 0.79, and the total score ranged from 4 to 20. Factor 2, supporting patient autonomy, included two items: The PAA can (1) reduce the pressure on family members when making decisions and (2) ensure that my medical treatment decisions are respected. The second factor had a Cronbach’s α value of 0.74, and the total scores ranged from 2 to 10. The third factor, contributing to the collective good, comprised three items: With the PAA, (1) futile medical care can be reduced; (2) healthcare providers are protected from lawsuits while providing the treatment that I prefer; and (3) inefficient medical resource usage can be reduced. Factor 3 had a Cronbach’s α value of 0.88, and the total scores ranged from 4 to 20.

### Control variables

Education level was defined as the highest diploma respondents obtained. Because most respondents had a college diploma, we coded 1 as college and above and 0 as high school and below.

Age was treated as a continuous variable.

Marital status could be single or never married, married, divorced, or cohabitating. Because of the small number of divorced or cohabitating respondents, we coded 1 as married and 0 as not married.

## Results

One hundred potential participants were approached during the study, and 82 filled out the questionnaire successfully. Because of the COVID-19 pandemic at the time of the survey, access to the hospital for face-to-face contact for filling out the questionnaire was denied, hindering the recruitment of more respondents. Of the 82 questionnaires collected, 2 had missing data and were excluded from the analysis, yielding an effective response rate of 80%. Table 1 presents the demographic characteristics of the respondents and gender differences in these characteristics. Of the study respondents, 52.5% were women, 87.6% had a college education or higher, and 40% resided in Taipei City (where the hospital was located). Approximately 41.3% of the respondents’ family members (the patients) were admitted to the ICU at the time of the interview. About one-third (32.5%) of the respondents were the patients’ parents, 22.5% were the patients’ children, and 19% were the patients’ spouses. No gender differences were observed in these demographic characteristics.

**Table 1:** Demographic Characteristics of the Family Members of Hospitalized Patients (N = 80)

Variables	All (N = 80)	%/SD	Men (N = 38, 47.5%)	%/SD	Women (N = 42, 53.5%)	%/SD	X2/Independent two-sample t-test	
**Age (mean)**	45.10yrs	12.07	47.1yrs	11.6	43.1yrs	11.9	1.52	ns
**Education level**								
High school and below	10	12.5	4	10.5	6	14.3	2.60(2)	ns
College	53	66.3	23	60.5	30	71.4		
Graduate school	17	21.3	11	28.9	6	14.3		
**Marital status**								
Single	22	27.5	9	23.7	13	31	3.55(3)	ns
Married	55	6.8	27	71.1	28	66.7		
Divorced	1	1.3	0	0	1	2.4		
Cohabitation	2	2.5	2	5.3	0	0		
**Respondent’s relationship with the patient**								
Spouse	15	18.8	6	15.8	9	21.4	8.92(6)	ns
Parents	26	32.5	10	26.3	16	38.1		
Children	18	22.5	12	31.6	6	14.3		
Siblings	3	3.8	3	7.9	0	0		
Friends	8	10	3	7.9	5	11.9		
Self	3	3.8	2	5.3	1	2.4		
Unspecified	7	8.8	2	5.3	5	11.9		
**Family monthly income**								
USD 0 ~ 1,011	7	8.9	3	8.1	4	9.5	2.93(2)	ns
USD 1,012 ~ 3,371	39	49.4	22	59.5	17	40.5		
USD 3,372 and above	33	41.8	12	32.4	21	50		
**Employment**								
Unemployed	4	5	2	5.2	2	4.8		
Retired	12	15	6	15.8	6	14,3	0.04(2)	ns
Yes	64	80	30	78.9	34	80.9		
**Place of residence**								
Taipei City	32	40	16	42.1	16	38.1	0.148(2)	ns
Outside Taipei City	46	57.5	21	55.3	25	59.5		
Foreign countries	2	2.5	1	2.6	1	2.4		
**Hospital ward**								
ICU	33	41.25	24	63.2	23	54.8	0.58(1)	ns
Internal/Surgical ward	47	58.75	14	36.8	19	45.2		

Rounding differences to 100% are possible

Gender differences were discovered across all three factors for attitude toward the PAA (Table 2). The men had higher average scores regarding the PAA promoting a sense of abandonment than the women (8.94 vs. 7.48; *p* < 0.05). However, the women had higher scores for supporting patient autonomy (8.74 vs. 7.94; *p* < 0.01) and contributing to the collective good (8.64 vs. 7.47, *p* < 0.001) than the men.

**Table 2:** Gender Differences in Attitude Toward the Patient Autonomy Act (PAA) (Mean, SD)

Attitude Items1	All	Men	Women	Independent two-sample t-test	
	Mean (SD)	Mean (SD)	Mean (SD)	T-value	Cronbach’s a
**Factor 1: Sense of abandonment**					0.79
With the PAA, I am basically waiting to die.	1.83 (0.81)	2.05 (0.73)	1.62 (0.83)	-2.47*	
With the PAA, I feel insecure about my medical treatments in the future.	2.29 (0.99)	2.45 (1.03)	2.14 (0.95)	-1.37	
With the PAA, my quality of care will not be guaranteed.	2.19 (0.86)	2.42 (0.83)	1.98 (0.84)	-2.38*	
With the PAA, healthcare providers will no longer care for my medical conditions.	1.88 (0.83)	2.03 (0.82)	1.74 (0.83)	-1.56	
**Sum scores (5–20)**	8.18 (2.73)	8.94 (2.46)	7.48 (2.81)	-2.48*	
**Factor 2: Support for patient autonomy**					0.74
PAA can reduce the pressure on family members to make decisions.	4.06 (0.80)	3.89 (0.80)	4.21 (0.78)	1.81	
PAA can make how I wish to be medically treated to be respected.	4.30 (0.70)	4.05 (0.70)	4.52 (0.63)	3.17**	
**Sum scores (5–10)**	8.36 (1.34)	7.94 (1.37)	8.74 (1.21)	2.74**	
**Factor 3: Collective good**					0.88
With the PAA, futile medical care can be reduced.	4.10 (0.88)	3.79 (0.91)	4.38 (0.76)	3.17**	
With the PAA, healthcare providers will be protected from lawsuits while treating me how I wish to be medically treated.	4.19 (0.66)	3.92 (0.59)	4.43 (0.63)	3.72***	
With the PAA, waste in medical resources can be reduced.	3.99 (089)	3.68 (0.96)	4.26 (0.73)	3.04**	
**Sum scores (5–15)**	8.08 (1.07)	7.47 (1.74)	8.64 (1.41)	3.32**	

1: The higher score, the stronger agreement with the statement

* P < 0.05; ** P < 0.01, *** P < 0.001

### Gender differences in the intention to withhold LSTs for severe dementia

As indicated in Table 3, compared to the women, the men were less likely to withhold LSTs for severe dementia (total scores, 15.84 vs. 18.88, *p* = 0.01). The men less likely to agree to withhold invasive LSTs even in case of severe dementia than were the women (enteral tube feeding, men vs. women 2.78 vs. 3.36, *p* = 0.05; dialysis, 2.66 vs. 3.43, *p* = 0.02; intubation with a ventilator, 3.23 vs. 3.93, *p* = 0.02; tracheotomy, 3.50 vs. 4.12, *p* = 0.01). Yet, men and women showed no difference in the intention to withhold resuscitation in case of a cardiac arrest (3.65 vs. 4.07, *p* = 0.2).

**Table 3:** Gender Differences in the Intention to Withhold LSTs involving Severe Dementia for Self and Self-others Differences (Mean, SD)

	Self			Self-parent differences^2^			Self-spouse differences^2^		
LSTs to be withheld	Men (N = 38)	Women (N = 42)	Independent two-sample t-test^1^	Men (N = 18)	Women (N = 24)	Independent two-sample t-test	Men (N = 15)	Women (N = 14)	Independent two-sample t-test
Enteral tube feeding as a means of ingesting food	2.78 (1.17)	3.36 (1.43)	p = 0.05	0.67 (1.19)*	0.54 (1.06)*	ns	0.50 (1.02)	0.60 (0.99)*	ns
Dialysis if have kidney failure	2.66 (1.19)	3.43 (1.36)	p = 0.01	0.60 (0.70)***	0.50 (0.78)**	ns	0.07 (0.83)	0.33 (1.05)	ns
To be intubated and utilizing a ventilator to breathe	3.23 (2.10)	3.90 (1.30)	p = 0.02	0.72 (1.02)***	0.75 (1.03)***	ns	0.07 (0.83)	0.20 (1.01)	ns
Undergo a tracheotomy If being intubated for a month and survival is not possible without a ventilator	3.50 (1.08)	4.12 (1.19)	p = 0.01	0.28 (0.75)	0.33 (0.64)*	ns	0.07 (0.73)	0.48 (0.74)*	ns
Undergo cardiopulmonary resuscitation if a cardiac arrest	3.65 (1.07)	4.07 (1.18)	ns	0.50 (0.79)**	0.08 (0.58)	p = 0.05	-0.07 (0.62)	-0.33 (0.72)	ns
**Total scores**	15.84 (4.91)	18.88 (5.60)	p = 0.01	-	-	-	-	-	-

1 Independent two-sample t-test was used to test gender differences

2 Pair-sample t-tests were used to test self-other differences by gender, and independent two-sample t-tests were used to test gender differences in self-other differences

* p < 0.05; ** p < 0.01; *** p < 0.001

### Gender differences in self–other differences in the intention to withhold LSTs for severe dementia

Regarding the intention to withhold LSTs for people close to the respondents, if those people had severe dementia, 42 (52.5%), 31 (38.8%), 3 (4%), and 4 (5%) of the respondents referred to parents, spouses, children, and friends, respectively, as people for whom they would make LSTs decisions. Table 3 summarizes the self–other differences in the respondents’ intention to withhold LSTs for severe dementia. The mean values indicate the differences between the respondents’ intention to withhold LSTs for themselves and those on behalf of their parents or spouses. A greater mean difference indicates greater discordance.

In terms of making hypothetical LST decisions for parents, both men and women tend to agree to treatments for their parents rather than themselves. The self–parent differences in the respondents’ decisions regarding enteral tube feeding was 0.67 (*p* = 0.05) for men and 0.54 (*p* = 0.05) for women, regarding dialysis was 0.60 (*p* = 0.001) for men and 0.50 (*p* = 0.01) for women, regarding intubation on a ventilator was 0.72 (*p* = 0.01) for men and 0.75 (*p* = 0.01) for women, and regarding tracheotomy was 0.28 (nonsignificant) for men and 0.33 (*p* = 0.001) for women. No gender differences were observed in these self–other differences. The only significant gender difference was noted for conducting CPR if a cardiac arrest because of end-stage dementia (self–parent difference = 0.50, *p* = 0.01 for men, and 0.08 [not significant] for women; gender difference, *p* = 0.05).

Regarding the intention to withhold LSTs for spouses, the hypothetical decisions men would make for themselves were consistent with those they would make for their spouses. However, the hypothetical decisions women would make for their spouses differed significantly from those they would make for themselves. The women were more likely to accept enteral tube feeding for their husbands than for themselves (self–spouse difference = 0.60, *p* = 0.05) and were more in favor of a tracheotomy for their husbands than for themselves in cases where survival appears unlikely without a ventilator (self–spouse difference = 0.48, *p* = 0.05). However, no significant gender differences in these self–spouse differences were observed.

### Testing for the mediation effect

We examined whether men and women differ in their attitudes toward the PAA and whether their attitudes affect their intention to withhold LSTs for severe dementia (the mediation model). Table 4 presents the regression results (“a” in Fig. 1) for gender on the three factors derived from the attitude toward the PAA (serving as three mediators in testing for the mediation effect). The results revealed that, in comparison with the women’s scores, the men’s scores were 1.55 higher for regarding the PAA as promoting a sense of abandonment (95% CI = 0.31, 2.80), 0.75 lower for supporting patient autonomy (95% CI = − 1.38, − 0.12), and 1.87 lower for contributing to the collective good (95% CI = − 2.89, − 0.84), when age, education, and marital status were controlled. None of the control variables were significantly associated with attitude toward the PAA.

**Table 4:** Mediators as Outcome Variables: The Gender Effect on the Attitude Toward Patient Autonomy Act (PAA)

	Sense of abandonment			Support for patient autonomy			Collective good		
**Variables**	B	SE	95% CI	B	SE	95% CI	B	SE	95% CI
**Gender (ref: Women)**									
Men	1.55	0.63	(0.31, 2.80)*	-0.75	0.3	(-1.34, -0.16)*	-1.77	0.48	(-2.73, -0.81)***
**Control variables**									
Age	0.001	0.03	(-0.06, 0.06)	-0.01	0.02	(-0.05, 0.02)	0.01	0.02	(-0.04, 0.05)
Education	-1.35	1.22	(-3.77, 1.07)	0.21	0.42	(-0.48, 1.32)	1.08	0.73	(-0.38, 2.54)
Married	-0.78	0.67	(-2.11, 0.56)	0.06	0.37	(-0.69, 0.80)	0.69	0.66	(-0.62, 1.99)
Constant	9.12	1.58	(5.98, 12.26)	8.94	0.96	(7.02, 10.85)	11.48	1.4	(8.70, 14.26)
Model R^2^	0.11			0.11			0.19		

* p < 0.05; ** p < 0.01; *** p < 0.001

Table 5 presents the regression results (“b,” “c,” and “ab” in Fig. 1). The direct effect of gender on the intention to withhold LSTs for severe dementia were fully mediated through the three factors of attitude toward the PAA, respectively. The indirect effect (“ab” in Fig. 1) of gender through the factor of regarding the PAA as promoting a sense of abandonment (Mediator 1) on the intention to withhold LSTs was − 0.77 (95% CI = − 1.96, − 0.034), and that through the factor of supporting patient autonomy (Mediator 2) is − 0.91 (95% CI = − 1.96, − 0.09), and that through the factor of contributing to the collective good (Mediator 3) was − 1.65 (95% CI = − 2.99, − 0.52). These findings suggest that in comparison with the women, the men were significantly less likely to withhold LSTs involving severe dementia (the total effect [“c” + “ab” in Fig. 1] of gender was − 3.37 [95% CI = − 5.90, − 0.85]).

**Table 5:** Mediation Models: The Direct, Indirect, and Total Effect of Gender (X) on the Intention to Withhold LSTs (Y) involving Severe Dementia

	Intention to Withhold LSTs (Y)								
	**Model 1 (Mediator 1)**			**Model 2 (Mediator 2)**			**Model 3 (Mediator 3)**		
	B	SE	95% CI	B	SE	95% CI	B	SE	95% CI
**Gender (X) effect**									
Direct effect of X on Y	-2.6	1.36	(-5.30, 0.10)	-2.46	1.38	(-5.21, 0.28)	-1.73	1.42	(-4.56, 1.11)
Indirect effect of through M on Y	-0.77	0.51	(-1.96, -0.034)*	-0.91	0.49	(-1.96, -0.09)*	-1.65	0.63	(-2.99, -0.52)*
Total Effect of X on Y	-3.37	1.27	(-5.90, -0.85)*	-3.37	1.27	(-5.90, -0.85)*	-3.37	1.27	(-5.90, -0.85)*
**Mediators**									
Sense of abandonment	-0.53	0.25	(-0.99, -0.01)*	-	-	-	-	-	-
Support for autonomy	-	-	-	1.52	0.58	(0.367, 2.687)*	-	-	-
Collective good	-	-	-	-	-	-	1.1	0.33	(0.44, 1.76)***
**Control variables**									
Age	0.06	0.07	(-0.07, 0.19)	0.08	0.06	(-0.05, 0.20)	0.05	0.06	(-0.10, 0.18)
Education	0.18	2.67	(-5.15, 5.51)	0.34	2.38	(-4.41, 5.09)	-0.16	2.38	(-0.07, 0.18)
Married	1.07	1.45	(-1.83, 3.97)	1.39	1.49	(-1.59, 4.37)	0.82	1.46	(-2.09, 3.72)
Constant	19.16	4	(11.19, 27.13)	3.87	4.91	(-5.90, 13.64)	3.94	4.06	(-4.21, 12.09)
Model R2	0.18			0.2			0.24		

* p < 0.05; ** p < 0.01; *** p < 0.001

As indicated in Table 5, the different aspects of attitude toward the PAA had distinct effects on the intention to withhold LSTs for severe dementia (“b” in Fig. 1). Regarding the PAA as promoting a sense of abandonment reduced the intention to withhold LSTs for severe dementia (− 0.53, 95% CI = − 0.99, − 0.01); however, regarding the PAA as supporting patient autonomy (1.52, 95% CI = 0.37, 2.69) and as contributing to the collective good increased the intention to withhold LSTs for severe dementia (1.1, 95% CI=0.44, 1.76).

## Discussion

The results reveal that men tend to perceive the PAA and the signing of ADs as the abandonment of patients. In contrast, women tend to have more positive attitudes toward the PAA and view the PAA as supporting patient autonomy and contributing to the collective good. Men were generally less likely to withhold LSTs for severe dementia than women. However, the gender differences in the intention to withhold LSTs for severe dementia were fully mediated by differences in attitude toward the PAA between genders. This study contributes to the literature by exploring the disparity in how men and women perceive the PAA, an innovative piece of legislation in Taiwan that addresses a topic that generally receives little attention in Asian countries.

It may be easy to understand that people choose not to receive LSTs when the survival period is very short. According to a study by Tang et al. in Taiwan in 2014, among 2452 terminal cancer patients, when they knew the prognosis, they were less likely to choose life-prolonging medical treatment[52]. Yet, when the survival period may last several years, such as dementia, whether to withhold LSTs to shorten life will reflect more on people’s value concerning what is meant by the quality of life. For example, patients with severe dementia rely on life-supporting devices and other people for daily nutrition and hygiene, resulting in severely impaired quality of life and poor dignity. Ultimately, attitudes toward patient autonomy and associated ideas such as the quality of life may determine LST-related decisions in real situations.

This study found that women were hesitant to agree to LSTs for severe dementia if these treatments were perceived to be futile. Moreover, women felt empowered because they believed that ADs can prevent unwanted life support and that healthcare practitioners would honor their ADs, consistent with the results from Perkins et al. [21]. By contrast, men felt disempowered and feared harm from the healthcare system. A recent review of studies conducted in China indicated that unfamiliarity with the concept of advance care planning and ADs is the main reason for people’s reluctance to complete an AD [53].

Furthermore, although both the women and men in this study generally agreed to more LSTs for their parents than for themselves, this tendency was stronger among the men, who were more likely than women to agree to CPR for their parents even if their hearts stopped because of end-stage dementia. However, when the men were making hypothetical decisions on behalf of their spouses, the self–other differences disappeared; that is, the men made the same decisions for themselves and their spouses. By contrast, the women’s hypothetical LST decisions exhibited self–spouse differences: they were more likely to agree to tube feeding and tracheotomy for their husbands than for themselves. Because of the small sample size, no significant gender differences in self–spouse differences were observed in the intention to withhold LSTs for severe dementia.

These findings can be situated among the relevant literature on self–other differences [27, 54]. The risk-as-feelings theory holds that subjective risk preferences are attenuated in surrogate decisions [55]; when people make decisions on behalf of others, they are less influenced by their interpretation of the risks involved. Other biasing factors, such as social norms, can determine individuals’ choices on behalf of others. When sons or daughters act as surrogate decision-makers for their parents of advanced age, they may place more weight on social norms than on the risk of compromising their parents’ quality of life. We speculate that the hypothetical LST decisions that adult children make for their parents are affected by the principle of filial piety emphasized in Asian societies. As a result, the respondents in the present study indicated that they would agree to more hypothetical LSTs for their parents than for themselves in cases where the patient’s quality of life would be severely affected because of end-stage dementia.

The integrated model of surrogate decision-making proposed by Tunney and Ziegler [29] may also help elucidate intergeneration and gender differences in surrogate decisions in cases where a patient’s quality of life is highly compromised because of severe dementia. According to that model, the surrogate decision-maker may consider what is in the recipient’s best interest (benevolent perspective), what they would do if they were the recipient (projected perspective), what they believe the recipient would choose (simulated perspective), and what serves their interests irrespective of the recipient’s wishes (egocentric perspective). Familiarity [29] and closeness [27] with the recipient affect people’s ability to engage in simulated perspective-taking. Most Taiwanese families are still strongly influenced by patriarchy and traditional social hierarchies; surrogate decisions made by adult children on behalf of their parents or by women on their spouses are expected to be different from those made for themselves. Therefore, when people make decisions on behalf of others, they tend to be less influenced by the interpretation of risks involved and more influenced by other biasing factors, such as patriarchal principles and socialized gender roles. Several studies using health insurance claims data in Taiwan found that men, as fathers and husbands, are more likely than women to receive aggressive EOL care even at the end stage of terminal cancer [12, 13].

The current study provides a new perspective on how socialized gender roles affect the intention to withhold LSTs involving severe dementia. However, some limitations are noted. First, the sample is not representative because we adopted a single-center design with convenience sampling. Second, the questions regarding attitudes toward the PAA and the hypothetical scenarios involving severe dementia in the questionnaire were developed specifically for this study; a more extensive survey must be conducted to validate the questionnaire. Third, the answers to the questionnaire may be affected by the patients’ conditions on the respondents’ attitudes toward the PAA was not assessed. Last, the small sample size call for caution in the findings.

## Conclusion

The men in this study tended to perceive the PAA as a sign that patients are being abandoned by the health system and exhibited less interest than the women in regarding the PAA as a collective good and a means of protecting patient autonomy. Gender differences in the intention to withhold LSTs were fully mediated by their attitude toward the PAA. Both men and women were more likely to withhold LSTs for themselves than for their family members. Men were also more likely to agree to CPR for their parents than themselves, even if a cardiac arrest because of end-stage dementia, compared with what women would do for their parents.

Finally, women were more likely to agree to enteral tube feeding and a tracheotomy for their husbands than for themselves. In contrast, men made consistent decisions for themselves and their spouses for these LST scenarios. The study results indicate that socialized gender roles may play a critical role in EOL decisions in Taiwanese society. Because people still rely on family members to make LST decisions in real-life situations, patient education and public support for patient autonomy should be strengthened, especially in Asian countries.

## Data Availability

The datasets used and/or analyzed during the current study are available from the corresponding author, Duan-Rung Chen, upon reasonable request (email: duan@ntu.edu.tw).

## Abbreviations

PAA: Patient Autonomy Act.
ADs: Advance Directives.
CPR: Cardiopulmonary Resuscitation.
DGBAS: Directorate-General of Budget, Accounting, and Statistics.
DNR: Do-Not-Resuscitate.
EOL: End-of-Life.
ICU: Intensive Care Unit.
LSPQ: Life Support Prevalence Questionnaire.
LSTs: Life-Sustaining Treatments.

## References

[1] Fried TR, Bradley EH, Towle VR, Allore H (2002). Understanding the treatment preferences of seriously ill patients. N Engl J Med.

[2] Silveira MJ, Kim SY, Langa KM (2010). Advance directives and outcomes of surrogate decision making before death. N Engl J Med.

[3] Teno JM, Gruneir A, Schwartz Z, Nanda A, Wetle T (2007). Association between advance directives and quality of end-of-life care: A national study. J Am Geriatr Soc.

[4] Perkins HS (2007). Controlling death: the false promise of advance directives. Ann Intern Med.

[5] Ministry-of-Health-and-Welfare RoC. The Patient Autonomy Act. Law & regulations database of the Republic of China. [Internet]. 2020 [cited 2020 Dec 24];Available from: https://law.moj.gov.tw/LawClass/LawAll.aspx?pcode=L0020189.

[6] Mackenzie C, Rogers W (2013). Autonomy, vulnerability and capacity: a philosophical appraisal of the Mental Capacity Act. Int J Law Context.

[7] Wolf SM, Boyle P, Callahan D, Fins JJ, Jennings B, Nelson JL (1991). Sources of concern about the patient self-determination act. N Engl J Med.

[8] Khosla N, Curl AL, Washington KT (2016). Trends in engagement in advance care planning behaviors and the role of socioeconomic status. Am J Hospice Palliat Medicine®.

[9] Yadav KN, Gabler NB, Cooney E, Kent S, Kim J, Herbst N (2017). Approximately one in three US adults completes any type of advance directive for end-of-life care. Health Aff.

[10] Kitzinger J, Kitzinger C. Increasing understanding and uptake of advance decisions in Wales. 2016.

[11] Ministry of Health and Welfare. Information Page for Advance Directives Taipei, Taiwan [Internet]. 2019;Available from: https://hpcod.mohw.gov.tw/HospWeb/.

[12] Chang CM, Wu CC, Yin WY, Juang SY, Yu CH, Lee CC (2014). Low socioeconomic status is associated with more aggressive end-of-life care for working-age terminal cancer patients. Oncologist.

[13] Chang TS, Su YC, Lee CC. Determinants for aggressive end-of-life care for oral cancer patients: a population-based study in an Asian country. Medicine 2015;94. 10.1097/MD.0000000000000460 PMC4602967 25634186

[14] DeMartino ES, Dudzinski DM, Doyle CK, Sperry BP, Gregory SE, Siegler M (2017). Who Decides When a Patient Can’t? Statutes on Alternate Decision Makers. N Engl J Med.

[15] Ruhnke GW, Wilson SR, Akamatsu T, Kinoue T, Takashima Y, Goldstein MK (2000). Ethical decision making and patient autonomy: a comparison of physicians and patients in Japan and the United States. Chest.

[16] Chan CW, Wong MM, Choi KC, Chan HY, Chow AY, Lo RS (2019). Prevalence, perception, and predictors of advance directives among Hong Kong Chinese: A population-based survey. Int J Environ Res Public Health.

[17] Douglas R, Brown HN (2002). Patients’ attitudes toward advance directives. J Nurs Scholarsh.

[18] Lin HM, Yang CL, Chen MM, Chiu TY, Hu WY (2011). In-patients’ willingness on and acceptance of promotion for signing of advance directives. Taiwan J Hosp Palliat Care.

[19] Taylor DM, Cameron PA (2002). Advance care planning in Australia: overdue for improvement. Intern Med J.

[20] Chang HW, Yen CH, Lin PC, Liu LF (2011). Perspectives on advance directives in outpatients of Department of Family Medicine in Changhua City. Taiwan J Hosp Palliat Care.

[21] Perkins HS, Hazuda HP, Cortez JD (2004). Advance Care Planning: Does Patient Gender Make a Difference?. Am J Med Sci.

[22] Wright AA, Mack JW, Kritek PA, Balboni TA, Massaro AF, Matulonis UA (2010). Influence of patients’ preferences and treatment site on cancer patients’ end-of-life care. Cancer.

[23] Liu LN, Chen CH, Liu TW, Lin YC, Lee SC, Tang ST (2015). Preferences for aggressive end-of-life care and their determinants among Taiwanese terminally ill cancer patients. Cancer Nurs.

[24] Smets T, Rietjens JA, Chambaere K, Coene G, Deschepper R, Pasman HR, et al. Sex-based differences in end-of-life decision making in Flanders, Belgium. Medical Care 2012;815–20. 10.1097/MLR.0b013e3182551747 22525616

[25] Sharma RK, Prigerson HG, Penedo FJ, Maciejewski PK (2015). Male-female patient differences in the association between end-of-life discussions and receipt of intensive care near death. Cancer.

[26] Rietjens JA, Deschepper R, Pasman R, Deliens L (2012). Medical end-of-life decisions: does its use differ in vulnerable patient groups? A systematic review and meta-analysis. Soc Sci Med.

[27] Batteux E, Ferguson E, Tunney RJ. Risk preferences in surrogate decision making. Experimental psychology 2017. 10.1027/1618-3169/a000371 PMC5683735 28922998

[28] Ziegler FV, Tunney RJ (2012). Decisions for others become less impulsive the further away they are on the family tree. PLoS ONE.

[29] Tunney RJ, Ziegler FV (2015). Toward a psychology of surrogate decision making. Perspect Psychol Sci.

[30] Sulmasy DP, Snyder L (2010). Substituted interests and best judgments: an integrated model of surrogate decision making. JAMA.

[31] Carroll AE, Saha C, Ofner S, Downs SM (2019). Valuing health for oneself versus one’s child or elderly parent. J Health Psychol.

[32] Li L, Nelson JE, Hanson LC, Cox CE, Carson SS, Chai EJ (2018). How Surrogate Decision-Makers for Patients With Chronic Critical Illness Perceive and Carry Out Their Role. Crit Care Med.

[33] Torke AM, Sachs GA, Helft PR, Montz K, Hui SL, Slaven JE (2014). Scope and outcomes of surrogate decision making among hospitalized older adults. JAMA Intern Med.

[34] Nakamura K, Kinugasa Y, Sugihara S, Hirai M, Yanagihara K, Haruki N (2018). Sex differences in surrogate decision-maker preferences for life-sustaining treatments of Japanese patients with heart failure. ESC heart failure.

[35] Cheng YH, Wang JJ, Wu KH, Huang S, Kuo ML, Su CH (2016). Do-not-resuscitate orders and related factors among family surrogates of patients in the emergency department. Support Care Cancer.

[36] Ni P, Zhou J, Wang ZX, Nie R, Phillips J, Mao J (2014). Advance directive and end-of-life care preferences among nursing home residents in Wuhan, China: a cross-sectional study. J Am Med Dir Assoc.

[37] Chen DR, Chang LY, Yang ML (2008). Gender-specific responses to social determinants associated with self-perceived health in Taiwan: A multilevel approach. Soc Sci Med.

[38] Lu YH, Yi CC. Employment and family status of women in a changing society: allocation of household chores. In: Proceedings of conference on the Chinese family and its ethics. Center of Chinese Studies Taipei; 1999. page 321–37.

[39] Wu S-C. A national profile of family caregivers of the disabled elderly people in Taiwan. Chinese Journal of Public Health (Taipei) 1999;44–53.

[40] Chen YM, Chiang TL, Chen DR, Tu YK, Yu HW, Chiu WY (2022). Differing determinants of disability trends among men and women aged 50 years and older. BMC Geriatr.

[41] Husain AF, Stewart K, Arseneault R, Moineddin R, Cellarius V, Librach SL (2007). Women experience higher levels of fatigue than men at the end of life: a longitudinal home palliative care study. J Pain Symptom Manag.

[42] Winter L, Parks SM (2012). The reluctance to burden others as a value in end-of-life decision making: a source of inaccuracy in substituted judgment. J Health Psychol.

[43] Defining end of. life in dementia: A systematic review - Bria Browne, Nuriye Kupeli, Kirsten J Moore, Elizabeth L Sampson, Nathan Davies, 2021 [Internet]. [cited 2022 Jun 19];Available from: https://journals.sagepub.com/doi/full/10.1177/02692163211025457. 10.1177/02692163211025457 PMC8637358 34137314

[44] Sclan SG, Reisberg B (1992). Functional assessment staging (FAST) in Alzheimer’s disease: reliability, validity, and ordinality. Int Psychogeriatr.

[45] Lee P. Investigating the association between health literacy and life-sustaining treatment preferences of residents in coastal area in midland of Taiwan. Department & Graduate Institute of Medical Education & Bioethics, College of Medicine, National Taiwan University. 2018;1–81.

[46] Birch D, Draper J (2008). A critical literature review exploring the challenges of delivering effective palliative care to older people with dementia. J Clin Nurs.

[47] Kollisch DO, Santulli RB, Bernat JL (2021). The limits of advance directives in maintaining autonomy in patients with advanced dementia. Am J Med.

[48] Hsu YH, Chou MY, Chen HM, Chang WC, Chu CS, Wang YC (2020). The trend of aggressive treatments in end-of-life care for older people with dementia after a policy change in Taiwan. J Am Med Dir Assoc.

[49] Faul F, Erdfelder E, Lang AG, Buchner A (2007). G* Power 3: A flexible statistical power analysis program for the social, behavioral, and biomedical sciences. Behav Res Methods.

[50] Erdfelder E, Faul F, Buchner A (1996). GPOWER: A general power analysis program. Behavior research methods. instruments & computers.

[51] Wu CY, Chen D, rung, Hung ST (2020). Knowledge and attitudes regarding the Patient Autonomy Act and behavioral intention regarding signing advance decision among in-patients’ family members. Taiwan Gong Gong Wei Sheng Za Zhi.

[52] Tang ST, Liu TW, Chow JM, Chiu CF, Hsieh RK, Chen CH (2014). Associations between accurate prognostic understanding and end-of-life care preferences and its correlates among Taiwanese terminally ill cancer patients surveyed in 2011–2012. Psycho-oncology.

[53] Zhang X, Jeong SYS, Chan S (2021). Advance care planning for older people in mainland China: An integrative literature review. Int J Older People Nurs.

[54] Batteux E, Ferguson E, Tunney RJ (2019). On the likelihood of surrogates conforming to the substituted judgment standard when making end-of-life decisions for their partner. Med Decis Making.

[55] Loewenstein DA, Argüelles S, Bravo M, Freeman RQ, Argüelles T, Acevedo A (2001). Caregivers’ judgments of the functional abilities of the Alzheimer’s disease patient: a comparison of proxy reports and objective measures. The Journals of Gerontology Series B: Psychological Sciences and Social Sciences.

